# Examining South Tyrol’s Experience: Digital Health Adoption and Workforce Issues in Implementing Italy’s Primary Care Reform Under Ministerial Decree No. 77/2022

**DOI:** 10.3390/epidemiologia5040057

**Published:** 2024-12-23

**Authors:** Christian J. Wiedermann, Angelika Mahlknecht, Verena Barbieri, Dietmar Ausserhofer, Barbara Plagg, Carla Felderer, Pasqualina Marino, Adolf Engl, Giuliano Piccoliori

**Affiliations:** Institute of General Practice and Public Health, Claudiana—College of Health Professions, 39100 Bolzano, Italygiuliano.piccoliori@am-mg.claudiana.bz.it (G.P.)

**Keywords:** D.M. 77/2022, primary care reform, frailty assessment, digital health, workforce challenges, South Tyrol, telemedicine, electronic health records (EHRs)

## Abstract

**Background:** Ministerial Decree (D.M.) 77/2022 aims to reform Italy’s primary care system by establishing community health centres and integrating digital tools to address healthcare access disparities and workforce shortages. This review focuses on frailty assessment tools, digital health innovations, and workforce challenges in the Autonomous Province of Bolzano, South Tyrol, emphasising interprofessional trust and collaboration issues. **Methods:** Using a narrative custom review approach guided by the SANRA checklist, this study synthesised findings from PubMed, official health websites, and regional surveys on frailty, workforce dynamics, interprofessional collaboration, and digital infrastructure in South Tyrol. **Results:** General practitioners (GPs) exhibited high professional motivation but expressed concerns about autonomy and administrative burdens in collaborative care models. Trust issues between GPs and specialists hinder workforce cohesion and care coordination, highlighting the need for structured inter-professional communication. Frailty assessments, such as the PRISMA-7 tool, identify over 33% of community-dwelling individuals aged 75 years and older as frail, necessitating targeted interventions. Digital health adoption, particularly electronic health records and telemedicine, is slow because of workforce shortages and infrastructure limitations. **Conclusions:** The successful implementation of D.M. 77/2022 in South Tyrol requires addressing workforce challenges, improving interprofessional trust, expanding digital infrastructure, and integrating frailty assessment findings into care strategies. These measures are critical for achieving a more resilient, equitable, and effective primary healthcare system.

## 1. Introduction

The Italian National Health Service (NHS) is structured into a highly decentralised, three-tiered system comprising the central, regional, and local levels, with primary care primarily managed by Local Health Districts. However, significant variability in care organisation and quality persists and is influenced by factors such as geography, population size, and regional policies [1,2]. In contrast to other European Union countries, where group practices are prevalent [3], Italian general practitioners (GPs) operate independently in solo practices, serving an average of 1147 patients each [4]. This fragmented model, in conjunction with an aging GP workforce, wherein a substantial proportion are over 55 years of age, presents obstacles to transitioning towards integrated care models. Coordination between GPs and other healthcare providers is frequently limited, contributing to inefficiencies in care delivery [5].

Efforts to promote collaboration, such as the establishment of multidisciplinary primary care units under National Law 189/2012, have achieved limited success [6]. The COVID-19 pandemic further exposed critical weaknesses in Italy’s primary healthcare system, highlighting its fragmentation and limited capacity to manage public health emergencies [7]. These challenges necessitated the development of the National Recovery and Resilience Plan (PNRR) and the implementation of Ministerial Decree No. 77/2022, which aims to decentralise care, enhance service integration, and utilise digital health technologies to improve primary care delivery and accessibility.

**Figure 1:**
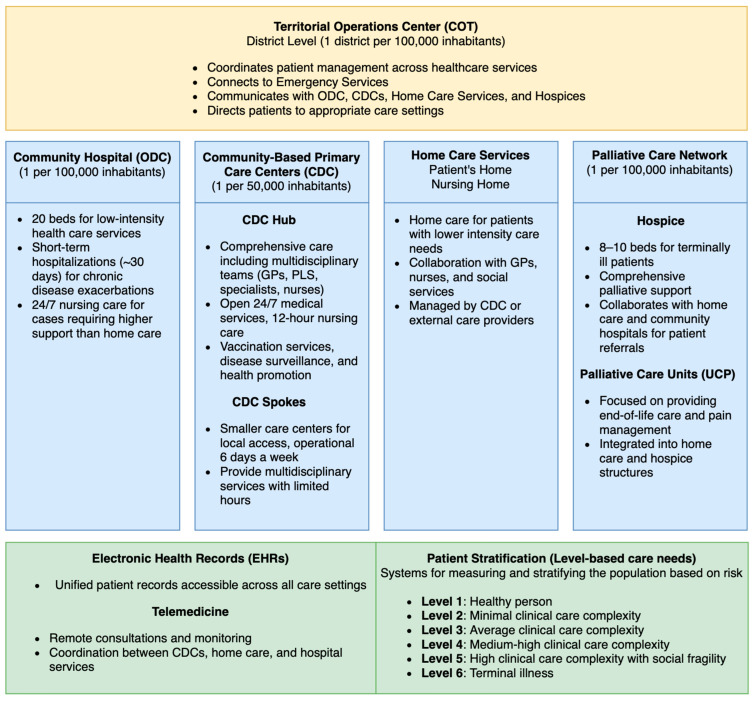
Structural organisation of territorial primary healthcare services introduced by D.M. 77/2022 in Italy. The reform emphasises the decentralisation of services into community-based primary care centres, community hospitals, and home care services coordinated by the Territorial Operations Center at the district level. Patient stratification defines various levels of care needs, from low-intensity services for healthy individuals to high-intensity services for terminally ill patients, and the integration of digital tools, such as Electronic Health Records and Telemedicine. Abbreviations: COT, territorial operations center (Centrale Operativa Territoriale); CDCs, community-based primary care centers (Case della Comunità); ODC, community hospital (Ospedale di Comunità); UCP, palliative care unit (Unità di Cure Palliative); EHRs, electronic health records.

D.M. 77/2022 focuses on restructuring primary healthcare through the establishment of Community Health Centers (Case di Comunità) and Community Hospitals (Ospedali di Comunità). These facilities aim to integrate health and social services, particularly for the underserved and at-risk populations. Frailty assessments play a crucial role in identifying vulnerable groups, thereby enabling targeted interventions to reduce hospitalisations. Furthermore, digital tools such as Electronic Health Records (EHRs) and telemedicine support care coordination and continuity, aligning with the reform’s objectives of improving equity and adaptability in the healthcare system [1,8] (Figure 1).

South Tyrol presents a unique setting for examining the implementation of D.M. 77/2022 due to its distinctive demographic and geographical characteristics [9]. The region exhibits a high proportion of elderly residents and significant disparities in healthcare accessibility between urban and rural areas [10]. These disparities manifest in variations in preventive health behaviours and outcomes [11,12]. To address these challenges, the reform aims to enhance the local healthcare infrastructure and integrate digital health solutions to improve access and equity. However, beyond the development of physical infrastructure, the reform faces critical obstacles in workforce development, particularly regarding the reluctance to transition from independent practices to collaborative care models. Overcoming these barriers is essential for reform to succeed in improving healthcare quality and accessibility across the region.

Effective primary healthcare reform necessitates addressing three interconnected challenges: frailty assessment, digital health integration, and workforce issues [8]. Frailty assessment is crucial for identifying at-risk populations, particularly in regions such as South Tyrol, with an aging population [11,12]. Proactive identification of frailty facilitates targeted interventions, thereby reducing hospitalisations and ensuring more equitable care. Similarly, digital health innovations, including telemedicine and EHRs, are essential to enhance care coordination and accessibility in geographically diverse regions. However, their adoption has been gradual, partly because of administrative and infrastructural limitations. Finally, workforce issues, including resistance among GPs to the transition from independent practices to collaborative care models, constitute a significant barrier to reform implementation [13,14,15]. Addressing these issues is imperative to achieving the objectives of D.M. 77/2022 and ensuring high-quality, sustainable healthcare services.

This article examines the implementation of D.M. 77/2022 in South Tyrol, focusing on three critical dimensions: frailty assessment, digital health integration, and workforce challenges. By exploring these interconnected themes, this study aimed to provide a comprehensive understanding of the barriers and opportunities in transitioning to a more integrated primary care system. The scope encompasses an analysis of survey data, case studies, and relevant literature, synthesising evidence to inform policy recommendations for improving healthcare equity, efficiency, and resilience in the region. To guide this analysis, this study addressed the following research questions:

- How can frailty assessment tools be effectively integrated into primary care to support proactive health care delivery?
- What are the key barriers to adopting digital health technologies in South Tyrol and how can they be overcome to enhance care coordination?
- How do workforce challenges, particularly GP resistance, impact the implementation of collaborative care models in D.M. 77/2022.

This study contributes to the extant literature on healthcare system reform by addressing lacunae in the comprehension of decentralised primary care model implementations. Utilising South Tyrol as a case study, this study presents novel perspectives on the convergence of frailty evaluation, digital health advancements, and workforce challenges. The research synthesises findings across these domains to provide empirically grounded recommendations for policymakers, underscoring the significance of evidence-driven strategies in realising the objectives of D.M. 77/2022. While the analysis primarily informs local reforms, it also yields insights applicable to other regions grappling with similar healthcare modernisation challenges.

## 2. Methods

**Figure 2:**
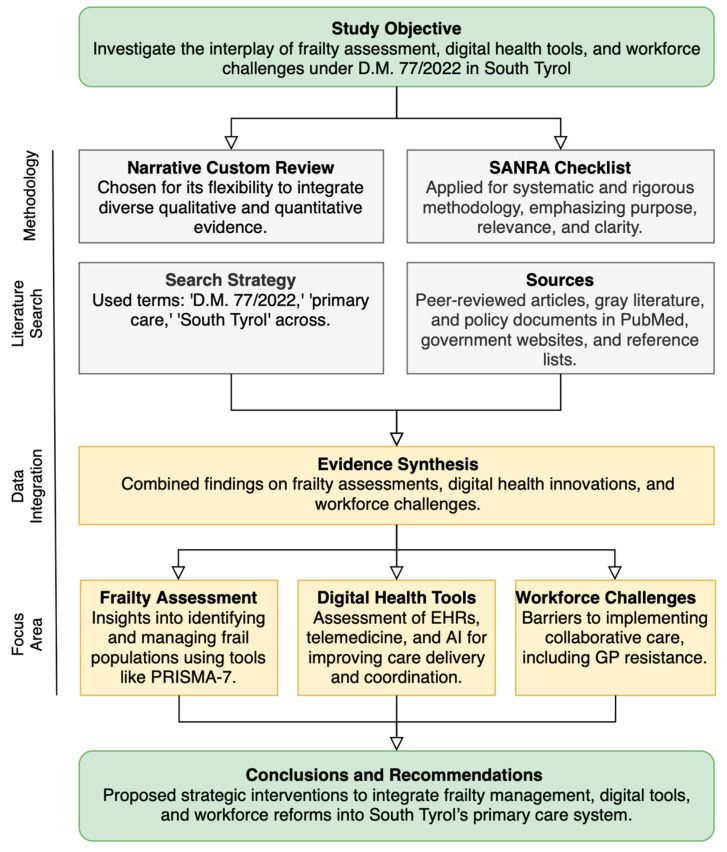
Framework for integrating frailty assessments, digital health tools, and workforce challenges under D.M. 77/2022 in South Tyrol. Abbreviations: AI, artificial intelligence; EHRs, electronic health records; D.M., ministerial decree (“Decreto Ministeriale”), PRISMA-7, Program on Research for Integrating Services for the Maintenance of Autonomy-7; SANRA, Scale for the Assessment of Narrative Review Articles.

Figure 2 presents an overview of the methodology. This study employed a narrative custom review methodology to address the multifaceted and interdisciplinary nature of healthcare reform. Custom reviews are uniquely suited to integrating diverse forms of evidence, both qualitatively and quantitatively, enabling a comprehensive synthesis of findings on frailty assessment, digital health innovations, and their interplay within the healthcare system in South Tyrol. Unlike traditional systematic reviews, which follow rigid inclusion and exclusion criteria, custom reviews offer the flexibility to incorporate a wide range of evidence types, including peer-reviewed articles, government reports, and grey literature. This adaptability allows for a holistic understanding of the complex challenges associated with implementing Ministerial Decree No. 77/2022. The methodology adheres to the guidelines of the Joanna Briggs Institute (JBI), which underscores the utility of custom reviews for topics requiring multidisciplinary analysis. By accommodating diverse evidence sources, the approach facilitates insights into how frailty assessments can inform proactive interventions and how digital health tools can address gaps in care delivery and accessibility [16].

The methodology was structured in accordance with the Scale for the Assessment of Narrative Review Articles (SANRA) checklist, ensuring a systematic and rigorous approach [17]. The SANRA checklist emphasises several key components that are central to this review: clarity of the purpose of the review, relevance of the selected literature, critical analysis of the included studies, and objective, well-supported conclusions. These principles were applied to ensure that the review maintained high methodological standards while allowing for flexibility in handling a range of data and insights from various healthcare domains.

The objective of the literature search was to identify all relevant literature pertaining to D.M. 77/2022, with particular emphasis on primary care reorganisation in South Tyrol, utilising diverse sources including PubMed and the official websites of the Italian Ministry of Health, as well as the reference lists of identified studies. The search terms employed encompassed “decreto ministeriale ‘OR “ministerial decree” AND “77/2022”,” “primary care’ OR “general practice”, and “South Tyrol ‘OR “Bolzano’ OR “Bozen ‘OR “Alto Adige”.’ The search was conducted without any temporal or linguistic restrictions. This encompassed peer-reviewed articles, government reports, and gray literature from healthcare institutions.

## 3. Results

### 3.1. Frailty Assessment

Frailty, a significant predictor of adverse health outcomes in elderly populations, requires early detection and tailored interventions to reduce hospital admissions and improve the quality of life [18]. Various regions have implemented improved frailty evaluation methods, including The Program on Research for Integrating Services for the Maintenance of Autonomy-7 (PRISMA-7) and Clinical Frailty Scale, to enhance care for this susceptible group [19].

In South Tyrol, frailty among the elderly is a significant concern, particularly given the region’s demographic profile, with a high proportion of older adults. The ASTAT survey, conducted in 2023, provides representative insights into frailty levels in the population aged >75 years [20]. Frailty was assessed using the PRISMA-7 tool, which classifies 33.9% of community-dwelling older adults as frail. These data are essential for shaping the implementation of D.M. 77/2022, as frail elderly populations require tailored interventions to ensure they can receive appropriate care in their community.

In addition to the findings from the ASTAT survey on frailty in South Tyrol, an ongoing study involving 19,501 patients aged >74 years was conducted by the GPs across the region. The primary objective of this study was to systematically register the level of frailty among community-dwelling elderly individuals using the PRISMA-7 screening tool, with a follow-up assessment using the Clinical Frailty Scale for patients with a score ≥3 on the PRISMA-7. GPs are central to large-scale projects, not only performing screenings but also coordinating care plans for frail individuals, including potential referrals to home care services based on Clinical Frailty Scale outcomes. The study involved voluntary participation with the data collection set to continue through 2024. The ASTAT survey indicates a significant need for targeted interventions in both urban and rural settings. This ongoing study will provide critical data to guide the implementation of D.M. 77/2022, particularly in shaping community-based care for South Tyrol’s aging population.

Given the scale of this study and its alignment with the goals of D.M. 77/2022, the results will be instrumental in understanding how frailty can be managed through the new primary care model, especially in rural areas where access to healthcare services is more limited.

### 3.2. Digital Health Innovations

Digital health technologies, including telemedicine, EHRs, and AI-based systems, possess considerable potential to enhance care delivery and coordination in South Tyrol, aligning with the objectives of D.M. 77/2022 to establish a more integrated and efficient primary healthcare system.

An ASTAT survey highlighted the digital readiness of this population in South Tyrol. Although many elderly individuals over the age of 74 in South Tyrol show limited engagement with digital tools, a significant portion (about 30%) use computers or tablets and around 40% use smartphones. The readiness to adopt more advanced digital health technologies such as telemedicine, wearables, and assistive robots varies, with 33.7% expressing openness to wearable devices and 30.1% willing to adopt telemedicine. These data are relevant for the ongoing digital transformation supported by D.M. 77/2022, as digital health tools will play a crucial role in monitoring and managing frailty and chronic conditions in older adults, allowing them to age in place while receiving continuous care.

These findings underscore the importance of enhancing digital literacy and access to technology among older adults to fully leverage the benefits of digital health interventions. Tailored education and support initiatives will be key to ensuring that these populations are not left behind in the transition to more integrated and digitally supported primary care models.

The adoption of digital health technologies, including telemedicine, EHRs, and artificial intelligence (AI)-based tools, holds promise for enhancing care delivery and coordination [21]. These tools support healthcare providers by improving diagnostic accuracy, enabling remote monitoring, and facilitating data sharing. However, evidence of improved diagnostic accuracy using AI in clinical settings remains limited.

#### 3.2.1. Chronic Disease Management

The integration of digital health tools, such as AI-based symptom checkers, presents a significant opportunity to enhance primary care services in D.M. 77/2022. According to a study conducted in South Tyrol, patients using symptom-checking AI tools were generally positive about the technology, appreciating its ease of use and the potential to save time during consultations [22]. These tools offer preliminary diagnostic insights that can empower patients and encourage them to manage their health more proactively [23]. Approximately half of the patients surveyed indicated a willingness to use such tools at home for initial health appraisals, potentially reducing unnecessary medical visits. GPs, on the other hand, exhibited more caution with concerns about the accuracy of AI diagnostics, the additional workload that these tools might introduce, the indispensability of personal contact, and the physical examination of patients. The GPs emphasised the importance of maintaining human expertise, particularly in complex cases, while acknowledging the potential of AI tools to assist in managing routine cases and relieving strain on the healthcare system [22]. This study highlights the need for the careful integration of these technologies with adequate clinical oversight.

The Assessment of Burden of Chronic Conditions (ABCC) tool provides an innovative framework for managing chronic diseases in line with the objectives of D.M. 77/2022. This tool facilitates continuous monitoring and personalized care, particularly for conditions such as diabetes, COPD and heart failure, which are prevalent among the aging population [24]. The ABCC tool supports a data-driven approach to chronic disease management, aligning with the goals of newly established community health centres and community hospitals, where managing complex chronic conditions is the primary focus. The ABCC tool demonstrates beneficial outcomes in terms of perceived care quality and patient engagement, indicating its readiness for implementation in clinical settings [25].

#### 3.2.2. Communication Tools and Telemedicine at the Primary-Secondary Care Interface

Telemedicine and remote monitoring platforms can assist healthcare providers in managing frail patients by delivering timely interventions and reducing hospital admissions [26]. The use of predictive models for risk stratification, as outlined in D.M. 77/2022, can further improve care by identifying high-risk individuals and enabling early interventions through digital platforms.

The successful implementation of D.M. 77/2022 in South Tyrol requires improved communication between GPs and hospital physicians. A recent survey of 313 participants (82 GPs and 231 hospital physicians) highlighted significant gaps in the existing communication infrastructure [15]. While basic tools such as phone and email are available, only approximately 53% of hospital physicians and 48% of GPs were satisfied with the current communication. Approximately one-third of the respondents reported difficulty in reaching their counterparts by phone, indicating a clear need for more reliable contact methods. To address these issues, the establishment of dedicated communication lines between GPs and specialists has been identified as critical. Over 74% of hospital physicians rated this as “very important” or “important”. Implementing these changes is essential to ensure smoother coordination and more efficient patient care, such as the South Tyrol transitions under D.M. 77/2022.

The survey also examined attitudes towards telemedicine and found that 73.6% of hospital physicians and 54.1% of GPs supported its expansion. However, GPs, particularly in rural areas, face barriers to adopting digital tools, highlighting the need for further investment in infrastructure and training [15].

Improving communication platforms and fostering greater telemedicine adoption will be pivotal for reform success, especially in rural areas. Immediate steps, including dedicated departmental contacts and better digital infrastructure, are necessary to enhance the integration of care between primary and secondary health care providers.

In the context of addressing the challenges posed by D.M. 77/2022, the South Tyrol General Practice Research Network (SAMNET) has emerged as a recent initiative to strengthen primary care through collaborative research and data-driven innovation. Established to enhance the quality of care and support the digital transformation of healthcare, SAMNET facilitates standardised data collection and fosters collaboration among GPs across the region. By integrating digital health tools such as the ABCC tool, SAMNET is actively contributing to the development of personalised care models and the management of chronic diseases, a central focus of community health centres (Case di Comunità). Moreover, SAMNET plays a pivotal role in promoting evidence-based approaches, supporting GPs in adopting digital innovations, and facilitating inter-professional collaboration, all of which are essential for the successful implementation of D.M. 77/2022. As a platform for continuous research and quality improvement, SAMNET’s objective is to ensure that the healthcare system in South Tyrol is prepared to meet the demands of reform by 2026.

### 3.3. Workforce Challenges

#### 3.3.1. GP Resistance and Trust Issues

The transition to the new primary care model under D.M. 77/2022 has faced notable resistance from GPs in Italy, including those in South Tyrol. The reluctance of GPs to transition into the new primary care model is a multifaceted issue heavily influenced by the reorganisation of healthcare roles and introduction of new healthcare infrastructure [27]. A significant concern is the shift from the current independent practice model to community health centres (Case di Comunità), which requires GPs to work more collaboratively in multidisciplinary teams. This transition has raised fears among GPs about losing professional autonomy, increasing the bureaucratic burden, and altering their traditional working structures. The added responsibility of coordinating with other healthcare professionals, including nurses and specialists, is perceived as a disruption to their practice and daily workflow [27]. Additionally, there is apprehension over managing dual responsibilities and balancing work in their own practices with the expected hours in community health centres.

Addressing these concerns requires targeted reforms in medical education and training to better prepare and incentivise young doctors to enter community-based settings [28]. The Italian healthcare system needs to provide a clearer career progression, improve working conditions, and foster a more collaborative approach to practice. Additionally, supporting GPs during the transition into these new centres is crucial to ensure the success of the reform.

Moreover, as the role of GPs is redefined under this reform, there is a strong emphasis on digital transformation and the integration of new technologies, such as telemedicine and EHRs. These changes, although seen as necessary for modernising healthcare, add to the perceived complexity of GPs’ roles of GPs. This resistance is further compounded by the fact that Italy’s public health system is already dealing with staff shortages and the aging GP workforce, which exacerbates concerns about increased workload.

A survey among healthcare professionals highlighted the fear that the new system, while beneficial in terms of infrastructure and resources, does not adequately address the workforce challenges critical to its successful implementation. More than 57% of respondents cited a shortage of personnel as the main obstacle, with 75% recognising that GPs would need to significantly adapt their approach to fit the new model [29].

To ensure the success of the reform, there is a pressing need to address these concerns through better medical workforce planning, improved training for GPs in managing multidisciplinary teams, and provision of clearer career pathways and financial incentives. The goal is to support GPs in transitioning to their new roles within the evolving healthcare framework while ensuring that patient care remains uninterrupted [29].

A subset of GPs has demonstrated resistance to the implementation of the novel collaborative care model as stipulated in D.M. 77/2022, expressing apprehensions regarding professional autonomy and increased administrative responsibilities. Notably, among practitioners approaching retirement age who have extensive experience in independent practice models, legitimate queries arise concerning the impact on their quotidian professional activities. These apprehensions underscore the imperative for supportive transition strategies that acknowledge the value of GPs’ experiences and insights. Addressing these concerns through transparent communication, appropriate incentives, and ongoing professional development opportunities may effectively leverage GPs’ expertise and position them as crucial agents for the transformation of primary care.

#### 3.3.2. Recruitment Challenges

Medical students from South Tyrol are often reluctant to return and practice as GPs in their home region after studying abroad because of several factors identified in a recent survey [30]. One prominent reason is the perceived lack of professional autonomy and career advancement opportunities in general practice, especially compared to specialist careers, which are seen as offering a more targeted expansion of medical knowledge, better financial rewards, and clearer career paths. Additionally, workload concerns, particularly the perception that GPs must handle numerous routine cases with insufficient time for each patient, contribute to reluctance. General practice training quality is also perceived as lower than specialist training quality, affecting students’ confidence in choosing it as a career. The poor reputation of general practice compared to other specialties further diminishes its appeal. Improving the attractiveness of general practice requires offering better undergraduate education with practical experience in primary care settings and ensuring that GPs maintain their professional autonomy. Additionally, measures to improve work-life balance and create more collaborative work environments could help address these issues and encourage more medical graduates to pursue general practice [30].

#### 3.3.3. Interprofessional Collaboration and Job Satisfaction

The workforce challenges in South Tyrol, particularly in primary care, are indicative of broader national trends, but encompass additional local complexities. GPs in South Tyrol have expressed significant concerns regarding their workload, professional autonomy, and coordination with secondary care providers. The findings of a recent cross-sectional survey elucidated the influence of relational coordination on job satisfaction and retention, revealing that inadequate interprofessional collaboration is a primary factor contributing to dissatisfaction, particularly among mid-career professionals [13,14].

In South Tyrol, bilingualism introduces an additional layer of complexity to the healthcare workforce, necessitating the effective communication of healthcare professionals across language groups. This factor, in conjunction with regional workforce shortages and an aging GP workforce, exacerbates the challenges of implementing D.M. 77/2022, which requires substantial coordination between multidisciplinary teams. The reluctance of GPs to transition from their independent practices to new community health centres is influenced by concerns regarding increased bureaucratic workloads and integration into salaried, team-based structures [15].

A 2019 survey of South Tyrolean GPs highlighted the critical factors influencing their professional motivation and areas requiring improvement within the healthcare environment [31]. Despite the increasing workload, GPs reported relatively high levels of motivation, with an average score of 7.0 out 10, especially among younger practitioners. This demonstrates a foundational commitment to patient care and an adaptability to evolving healthcare demands. However, the survey also revealed distinct stressors, including strain from demanding patients and a perceived lack of appreciation for services provided without compensation. The survey identified several priority areas for improvement that align with D.M. 77/2022’s objectives to build a more resilient and sustainable healthcare workforce. These include enhancing collaboration with specialists and healthcare providers across the territory, increasing GPs’ involvement in decision making, and reducing bureaucratic and administrative burdens that impede clinical efficiency. Additionally, there is a pressing need for improved public understanding and recognition of the value that general medicine provides in the healthcare system. Furthermore, the 2019 survey underscored the importance of compensating GPs for additional services and specialised skills to sustain motivation and retention. The respondents called for structured incentives to reflect the complexity of certain healthcare tasks such as home visits and emergency care, particularly in rural and underserved regions. Supporting flexible working arrangements and group practice settings have emerged as crucial factors for attracting younger GPs and fostering a collaborative environment within primary care.

Additional survey data show that GPs in South Tyrol report lower satisfaction with referral systems, emphasising gaps in communication between primary and secondary care [13,15]. Furthermore, the introduction of digital health tools such as EHRs has been slow, with professionals expressing concern over the administrative burden associated with these tools [15]. Addressing these workforce issues is crucial for the successful integration of the new model, as proposed under D.M. 77/2022, and requires targeted interventions aimed at improving communication, job satisfaction, and digital tool adoption [13,14].

This context underscores the necessity of workforce development strategies that encompass enhanced training for multidisciplinary collaboration, incentives for GP retention, and digital upskilling to ensure the efficacy of reform in South Tyrol. These findings highlight the requirement for targeted reforms that not only address infrastructure and workforce distribution but also support GPs’ professional well-being and autonomy. Enhancing the appeal of general practice through recognition, incentives, and interprofessional collaboration is crucial for the successful implementation of D.M. 77/2022 in South Tyrol.

## 4. Discussion

The implementation of D.M. 77/2022 in South Tyrol elucidates the interdependence of three critical components: frailty assessments, digital health tools, and workforce reforms. Addressing the challenges within these domains is imperative to establish an integrated and resilient primary care system.

### 4.1. Interconnected Challenges in Healthcare Reform

#### 4.1.1. Digital Infrastructure Gaps

The inadequate implementation of digital health technologies such as Electronic Health Records (EHRs) and telemedicine has hindered collaboration among healthcare professionals in South Tyrol. Research indicates that insufficient digital infrastructure impedes timely communication and evidence-based care delivery, thereby exacerbating inefficiencies across care levels [32]. To address this issue, robust EHR systems and telemedicine platforms must be deployed expeditiously in conjunction with comprehensive training programs for healthcare providers. These tools are fundamental to the integration of frailty assessments and chronic disease management into routine primary care.

#### 4.1.2. Coordination Between Primary and Secondary Care

Persistent gaps in the communication between GPs and hospital physicians continue to impede care coordination. Surveys conducted in 2019 and 2023 indicated ongoing dissatisfaction with communication modalities, such as telephone and electronic mail [15], and a lack of feedback regarding referral appropriateness [31]. This deficit in trust between GPs and specialists contributes to occupational dissatisfaction, reduced workforce retention, and inefficient patient-care pathways. To address these gaps, the establishment of centralised communication hubs within community health centres and the utilisation of telemedicine for interprofessional consultations are essential. Regular meetings, structured communication platforms, and inclusive decision-making processes can foster trust, enhance collaboration, and optimise patient management.

#### 4.1.3. Integrating Frailty and Chronic Disease Management

The management of frailty and chronic diseases in South Tyrol’s aging population necessitates the comprehensive integration of predictive analytics and frailty screening tools (for example, PRISMA-7 [20] and Clinical Frailty Scale) and chronic disease management systems such as the ABCC tool [24,25]. Current initiatives demonstrate potential, but remain fragmented, with incomplete integration into digital health platforms. Predictive models, in conjunction with remote monitoring technologies, can facilitate targeted interventions, thereby reducing hospitalisation and enhancing care quality. The expansion of telemedicine for the management of frail and chronically ill patients is crucial to achieve the objectives outlined for D.M. 77/2022.

#### 4.1.4. Workforce Shortages and Resistance to Change

The resistance of GPs to transition into multidisciplinary Community Health Centres (Case di Comunità) poses a significant barrier to reform. This reluctance is deeply rooted in the traditional model of general practice, where GPs worked autonomously, often for decades, maintaining exclusive relationships with their patients. These long-standing patient relationships, combined with sole responsibility for patient records and decision-making, have shaped a professional culture that is resistant to sharing responsibilities or adopting collaborative care frameworks. Many GPs, particularly older practitioners, view the transition to a team-based model as a threat to their professional autonomy and to the personalised care they have historically provided.

Compounding these challenges concerns increased administrative burdens and the shift to salaried employment models, which are perceived as undermining independence. Additionally, South Tyrol faces significant workforce shortages, particularly in rural and bilingual regions, where the demands on GPs are already heightened because of dual-language requirements and geographic isolation [30].

Modern societal changes have further exacerbated the need for reform. A growing focus on work-life balance has shifted expectations among younger GPs, who are less willing to adopt the 24/7 availability traditionally associated with the profession. The current workforce structure is increasingly seen as unsustainable, with both older and younger GPs acknowledging the strain of balancing professional demands with personal commitment.

### 4.2. Building an Integrated Healthcare System

The interplay between frailty assessments, digital tools, and workforce reforms underlines the need for an integrated approach to healthcare reform. Systematic assessments of prefrailty and frailty require robust digital platforms to enable predictive analytics and seamless coordination between primary and secondary care providers [33].

The adoption of digital health technologies, including EHRs and telemedicine platforms, facilitates better integrated care across different levels and providers and enhances care coordination and patient outcomes [34]. Digital health tools require a well-trained and motivated workforce to ensure their adoption and effective utilisation in clinical practice. However, the successful implementation of these digital tools is contingent on a well-trained and motivated workforce. Currently, numerous healthcare professionals lack awareness and training in frailty screening, assessment, and management.

Educational programs have demonstrated efficacy in enhancing healthcare professionals’ knowledge of frailty and their self-reported competence in assessing frailty, emphasising the necessity for comprehensive training initiatives [35].

Workforce reforms should prioritise collaborative care models that integrate digital innovation and frailty management strategies. Multidisciplinary team (MDT) work is essential for optimising and integrating services for people who are frail. However, many healthcare and social care professionals have not received formal training in collaborative work. Implementing MDT training designed to help participants deliver integrated care for frail individuals can enhance collaboration and improve patient outcomes [36].

### 4.3. Recommendations

Guided by the research questions outlined in the Introduction, the recommendations aim to provide insights into overcoming these barriers while leveraging the strengths of the regional healthcare system.

#### 4.3.1. Digital Infrastructure and Care Coordination

The integration of frailty assessments and chronic disease management tools into primary care depends on a robust digital infrastructure [33,37]. However, the current slow adoption of EHRs and telemedicine platforms in South Tyrol limits the potential for effective care coordination between primary and secondary care providers [32]. Interventions should focus on expanding digital systems that incorporate telematic solutions for efficient access to hospital records and diagnostic findings alongside targeted training programs to equip healthcare professionals with the necessary digital skills. These digital tools can significantly improve the efficiency and quality of healthcare delivery by facilitating real-time access to patient data and enhancing inter-professional communication.

#### 4.3.2. Interprofessional Communication and Collaboration

Effective communication between GPs and hospital physicians is crucial for seamless care transition and patient management [38,39]. Surveys conducted in South Tyrol indicate substantial dissatisfaction with current communication tools, with minimal improvements observed between 2019 and 2023 [15,31]. Dedicated communication platforms and centralised communication hubs within community health centres are essential for addressing this issue. Enhanced telemedicine capabilities can further facilitate consultations and referrals, supporting trust and collaboration among healthcare professionals. These measures directly addressed the second research question by addressing the key barriers to adopting digital tools that facilitate care coordination.

#### 4.3.3. Integration of Frailty and Chronic Disease Management Tools

Proactive management of frailty and chronic diseases is central to DM success. 77/2022 [40,41]. While tools such as PRISMA-7 and the Clinical Frailty Scale are already in use, their integration with digital platforms remains incomplete, limiting their potential to drive targeted interventions and reduce hospitalisations [20,25]. Although promising for chronic disease management, the ABCC tool requires further evidence of its clinical effectiveness before large-scale implementation. Expanding the use of predictive analytics and telemedicine for risk stratification and remote monitoring is crucial for addressing the needs of frail and chronically ill patients, aligning healthcare delivery with D.M. 77/2022 objectives.

#### 4.3.4. Workforce Challenges and Resistance to Change

The effective implementation of collaborative care models necessitates overcoming resistance from GPs, particularly those accustomed to autonomy in traditional practice structures. Studies have shown that GPs highly value their professional independence, and any perceived threat to this autonomy can lead to a reluctance to adopt new collaborative approaches. For instance, research indicates that GPs are motivated to engage in new models of collaboration when they respect their autonomy and enhance patient care. Conversely, concerns about losing control over clinical decisions can hinder willingness to participate in such models. Respecting GP concerns about autonomy and fostering interprofessional respect are crucial steps in successfully implementing collaborative care models [42]. Workforce shortages, especially in rural and bilingual regions of South Tyrol, exacerbate these challenges [30]. Addressing these issues requires a comprehensive approach, which includes the following:

- Incentives for GP Participation: Monetary remuneration, professional advancement opportunities, and adaptable employment models that equilibrate autonomy with collaboration in multidisciplinary teams.
- Recruitment and Retention Strategies: Focused initiatives to attract healthcare professionals to underserved areas and ensure a sustainable workforce in the future.
- Training Programs: Equipping emerging healthcare professionals with the requisite skills to excel in team-based care models and digital health environments.

By focusing on these interventions, South Tyrol could overcome the resistance to change identified in the third research question, ensuring that GPs are supported throughout the transition process.

#### 4.3.5. Synthesis of Recommendations

These recommendations demonstrate how frailty assessments, digital health tools, and workforce reforms are interdependent in shaping the future of primary care under D.M. 77/2022. Table 1 provides a comprehensive summary of these challenges, the current situation in South Tyrol, and proposed interventions. This table facilitates a clear understanding of how these measures address the gaps identified in the findings, offering a roadmap for achieving a resilient and effective healthcare system by 2026.

### 4.4. Future Studies on D.M. 77/2022 Implementation in South Tyrol

Two ongoing studies led by the Institute of General Practice and Public Health in Bolzano are relevant for understanding and addressing the healthcare challenges posed by D.M. 77/2022. These studies provide insights into both emergency care utilisation and the needs of caregivers in South Tyrol, directly informing healthcare reforms.

The first study, “The Care Accessibility and Reasons for Non-urgent Emergency Department Visits in South Tyrol” (CARES) (ISRCTN17355506), focused on identifying the underlying causes of non-urgent emergency department (ED) visits. This cross-sectional study aimed to determine how many such visits could be managed in primary care settings, thereby reducing unnecessary strain on ED resources. By examining the sociodemographic and clinical factors associated with these visits, the study highlights potential barriers to primary care access, such as GP availability or patient dissatisfaction with community-based services. A key factor also includes patients’ preferences for immediate specialist consultations or diagnostics, which are often subject to long wait times in the current system. Understanding these barriers is crucial for D.M. 77/2022, as one of its primary goals is to reduce hospital admissions through enhanced primary care infrastructure, particularly within community health centers (Case di Comunità). The findings from the CARES study will help to identify targeted interventions to improve access to community care and reduce avoidable ED visits, aligning with the broader aims of the decree.

The second study, “Factors Influencing the Care Burden and Needs Analysis of Caregivers of People with Dementia in Home Care” (LIFEZ) (ISRCTN19637698), investigates the care needs and psychological stress experienced by informal caregivers of dementia patients in South Tyrol. This study is relevant to D.M. 77/2022, which emphasises the integration of social and healthcare services to support vulnerable populations, such as the elderly and the chronically ill. The findings will provide critical data on how to better support family caregivers, enabling people with dementia to remain in their homes longer, while reducing caregiver burnout. Additionally, this study explores the accessibility of existing services, such as home care and emergency services, which are critical in regions such as South Tyrol, with its rural and geographically dispersed populations. The results will guide policymakers in how to better integrate home care services with new community health centres, ensuring that the needs of both patients and their caregivers are adequately addressed.

Both studies offer essential data that can inform the successful implementation of D.M. 77/2022 in South Tyrol by addressing gaps in service provision, access to care, and the support of vulnerable populations. They underscored the importance of integrating research findings into health policy and conducting outcome evaluations to ensure that future reforms are data-driven and target the most pressing healthcare challenges.

## 5. Conclusions

The implementation of D.M. 77/2022 constitutes a significant advancement in the modernisation of Italy’s primary healthcare system, particularly in addressing demographic challenges, healthcare disparities, and the integration of novel care models. In South Tyrol, these endeavours are crucial for addressing the unique regional challenges posed by an ageing population, geographic isolation, and linguistic diversity. However, the successful realisation of the reform objectives by 2026 faces substantial obstacles that necessitate urgent and targeted intervention.

This review elucidates the interconnectedness between three critical domains: frailty assessment, digital health tools, and workforce reforms. These domains are fundamental to establishing a patient-centred and integrated primary care system; however, each presents distinct challenges. Resistance among GPs to collaborative care models remains a significant impediment rooted in long-standing practices of professional autonomy and individual patient responsibility. In conjunction with workforce shortages and an aging healthcare workforce, particularly in rural and bilingual regions, these factors complicate the proposed timelines and goals of the reform.

Digital infrastructure is another critical area that requires substantial investment. The limited adoption of EHRs, telemedicine platforms, and other digital health tools constrains the potential for care coordination, frailty assessment, and chronic disease management. In the absence of comprehensive training programs and the integration of these tools into routine clinical practice, reform risks fail to achieve their intended impact. The integration of digital solutions must be concomitant with workforce preparedness, as healthcare professionals require the requisite knowledge and motivation to effectively utilise these technologies.

Frailty assessments, identified as a priority by D.M. 77/2022, underscore the necessity for proactive management of aging populations. Although instruments such as PRISMA-7 and the Clinical Frailty Scale have demonstrated potential, their fragmented integration into clinical workflows remains a challenge. Aligning these tools with digital platforms and predictive analytics can facilitate early interventions and reduce the burden on secondary care but only if supported by a cohesive implementation strategy.

The ambitious target of achieving full implementation by 2026 warrants re-evaluation, considering these persistent challenges. Workforce issues necessitate sustained efforts that extend beyond this timeframe, including enhanced recruitment strategies, improved working conditions, and incentives for young health care professionals to enter general practice. Structural changes in education and training are imperative to ensure that the next generation of GPs is equipped to embrace collaborative care models and digital innovations.

In conclusion, while D.M. 77/2022 offers a transformative vision for healthcare in South Tyrol, achieving its goals will require adaptive strategies, ongoing investment, and a realistic understanding of the complexities involved. Greater emphasis on workforce support, digital integration, and the phased rollout of new care models is critical for building a resilient and sustainable healthcare system that meets the needs of the population.

## Figures and Tables

**Table 1:** Challenges, strengths, and weaknesses of the implementation of D.M. 77/2022 in South Tyrol and the proposed interventions.

Category	Challenges	Situation in South Tyrol	Proposed Interventions
Digital Infrastructure	Slow adoption of EHRs and telemedicine platforms. Insufficient digital infrastructure to support care coordination.	Strengths: - Growing awareness and interest in digital tools Weaknesses: - Digital infrastructure underdeveloped - Limited use of telemedicine - Low adoption of EHRs by healthcare professionals	Invest in digital infrastructure expansion across all care settings Equip healthcare providers with necessary digital skills Promote EHR adoption and ensure telemedicine integration
Workforce	GP resistance to transitioning from independent practices to collaborative care models Workforce shortages, particularly in rural and bilingual regions	Strengths: - High commitment among local healthcare providers to improve primary care Weaknesses: - High percentage of aging GPs - Reluctance to adopt new team-based models - Shortage of bilingual staff	Provide career pathways, incentives, and professional autonomy to encourage participation Implement recruitment strategies, especially in rural and bilingual areas Foster GP retention through work-life balance improvements
Communication and Coordination	Lack of effective communication channels between GPs, hospital physicians, and other healthcare providers Gaps in care coordination across levels of care	Strengths: - Emerging efforts to improve interprofessional collaboration Weaknesses: - Dissatisfaction with current communication platforms - Poor interprofessional communication	Implement dedicated communication platforms for GPs and specialists Establish centralized communication hubs Enhance telemedicine use for consultations
Frailty and Chronic Disease Management	Integration of frailty assessments and chronic disease management tools is incomplete	Strengths: - Existing studies assessing frailty (e.g., PRISMA-7) Weaknesses: - Limited integration of frailty tools with digital health platforms - Lack of comprehensive chronic disease management tools	Integrate frailty and chronic disease management tools like ABCC with digital platforms Use predictive analytics for risk stratification Expand remote monitoring and telemedicine services for frail and chronically ill patients

Abbreviations: ABCC, Assessment of Burden of Chronic Conditions; EHRs, electronic health records; GP, general practitioner; PRISMA-7, The Program on Research for Integrating Services for the Maintenance of Autonomy-7.

## Data Availability

No new data were created.
